# The impact of internal-generated contextual clues on EFL vocabulary learning: insights from EEG

**DOI:** 10.3389/fpsyg.2024.1332098

**Published:** 2024-02-02

**Authors:** Weichen Zhou, Xia Wu

**Affiliations:** ^1^School of Teacher Education, Shaoxing University, Shaoxing, China; ^2^Department of Psychology, Shaoxing University, Shaoxing, China

**Keywords:** online EFL vocabulary learning, contextual clues, mental efforts, attentional engagement, EEG

## Abstract

With the popularity of learning vocabulary online among English as a Foreign Language (EFL) learners today, educators and researchers have been considering ways to enhance the effectiveness of this approach. Prior research has underscored the significance of contextual clues in vocabulary acquisition. However, few studies have compared the context provided by instructional materials and that generated by learners themselves. Hence, this present study sought to explore the impact of internal-generated contextual clues in comparison to those provided by instructional materials on EFL learners’ online vocabulary acquisition. A total of 26 university students were enrolled and underwent electroencephalography (EEG). Based on a within-subjects design, all participants learned two groups of vocabulary words through a series of video clips under two conditions: one where the contexts were externally provided and the other where participants themselves generated the contexts. In this regard, participants were tasked with either viewing contextual clues presented on the screen or creating their own contextual clues for word comprehension. EEG signals were recorded during the learning process to explore neural activities, and post-tests were conducted to assess learning performance after each vocabulary learning session. Our behavioral results indicated that comprehending words with internal-generated contextual clues resulted in superior learning performance compared to using context provided by instructional materials. Furthermore, EEG data revealed that learners expended greater cognitive resources and mental effort in semantically integrating the meaning of words when they self-created contextual clues, as evidenced by stronger alpha and beta-band oscillations. Moreover, the stronger alpha-band oscillations and lower inter-subject correlation (ISC) among learners suggested that the generative task of creating context enhanced their top-down attentional control mechanisms and selective visual processing when learning vocabulary from videos. These findings underscored the positive effects of internal-generated contextual clues, indicating that instructors should encourage learners to construct their own contexts in online EFL vocabulary instruction rather than providing pre-defined contexts. Future research should aim to explore the limits and conditions of employing these two types of contextual clues in online EFL vocabulary learning. This could be achieved by manipulating the quality and understandability of contexts and considering learners’ language proficiency levels.

## 1 Introduction

In recent years, online learning has gained popularity among self-directed learners due to its convenience and accessibility (Alzahrani, 2022). This flexible approach accommodates a wide range of subjects in today’s era of lifelong learning (McAuley et al., 2010). Online English vocabulary learning is particularly favored for its crucial role in global communication (Hyunjeong and Mayer, 2018; Lee and Harris, 2018). English as a Foreign Language (EFL) learners, especially those with limited proficiency, prefer online vocabulary learning for its self-paced nature (Arispe and Blake, 2012). Vocabulary is progressively learned through mobile applications and online platforms that provide multimedia content for enhanced learning (Chen et al., 2018; Roy et al., 2019; Wang et al., 2021). As a result, EFL vocabulary learning in this digital age has shifted from traditional paper-based methods to online multimedia environments (Alhatmi, 2023).

The question of how to effectively learn vocabulary online and optimize learning outcomes has drawn significant attention from researchers and educators (Mahmoudian, 2017; Yeh and Lan, 2018; Xu et al., 2020). It has long been thought that vocabulary acquisition should be determined by mechanical repetition rather than a deeper understanding of word meanings. Researchers used to suggest that vocabulary could be retained through continuous repetition, transferring words from short-term to long-term memory (Atkinson and Shiffrin, 1968; Gairns and Redman, 1986). However, contemporary research highlights the importance of understanding the relationships between target words and contextual clues for EFL learners (Wang and Huang, 2017; Fukushima, 2019). It is now understood that contextual clues, such as words, phrases, or sentences in text, play a pivotal role in aiding learners in associating unfamiliar words with their prior knowledge, a crucial step in vocabulary acquisition (Lowell et al., 2020; Jabar and Mansor, 2021). They serve as a vital cue for the indispensable semantic processing phase of vocabulary learning (Xu, 2010). However, the impact of contextual clues on EFL vocabulary learning, especially in online environments, warrants further exploration.

### 1.1 The advantage of learning EFL vocabulary online

Online vocabulary learning is beneficial for EFL learners primarily because digital materials provide multiple channels of information, enabling learners to make more effective use of their cognitive resources for meaningful learning (Mayer, 2017; Wolf, 2018). An increasing body of evidence suggests that exposure to digital language materials significantly enhances learners’ vocabulary comprehension and acquisition when compared to traditional printed materials (Bui et al., 2020; Cong-Lem and Lee, 2020). For instance, video lectures, as a prominent form of online instructional resource, are highly favored by EFL learners due to their inherent advantages (Ramezanali and Faez, 2019; Kokoc et al., 2020; Wang and Lee, 2021). Video captions effectively synchronize audio-visual input channels and guide learners’ attention, promoting deeper word processing and vocabulary acquisition (Montero Perez et al., 2015, 2018; Teng, 2019; Wang, 2019; Ouyang et al., 2020). Additionally, the presence of vivid instructor images in videos facilitates EFL learners’ vocabulary mastery through social cues like gestures, promoting interaction and motivation (Drijvers et al., 2019; Andra et al., 2020; Zhu et al., 2022) and delivering extra semantic information for vocabulary comprehension in an efficient manner (Drijvers and Ozyurek, 2016; Pi et al., 2021).

In addition to the inherent attributes of online materials, online vocabulary learning provides extensive communication opportunities for EFL learners through virtual chat rooms and network groups (Stashko, 2019). Increased interaction enhances learners’ motivation and self-perception as capable speakers (Skidmore, 2023), leading to satisfactory vocabulary acquisition through active participation and the use of social networking tools (Polat et al., 2013; Teng et al., 2022). These social benefits extend to other forms of online vocabulary learning, including digital games and virtual reality (Acquah and Katz, 2020; Huang et al., 2022). Besides, these innovative methods reduce language anxiety by creating a supportive social environment and enhancing learners’ autonomy through real-time interactivity (Jabbari and Eslami, 2019; Tseng et al., 2019; Tai et al., 2022). Consequently, learners gain confidence in their vocabulary development due to increased engagement (Calvo-Ferrer, 2017).

### 1.2 Research regarding contextual clues in EFL vocabulary learning

Contextual clues play a crucial role in vocabulary instruction, aiding learners in comprehending new words and grasping their semantic meanings (Wallace, 1982). Existing research suggests two ways of accessing contextual clues for semantic processing in vocabulary learning. First, related contexts can be provided by learning materials, such as example sentences accompanied by unknown words (Liu and Mostow, 2013). Example sentences with translations in the learners’ native language act as valuable scaffolding, especially for EFL learners with lower language proficiency (Jimenez and Kanoh, 2012; Pauwels, 2012). This promotes comprehensive vocabulary acquisition and facilitates subsequent review (Cheng and Good, 2009). Accordingly, researchers have attached great importance to the role of contextual clues provided by examples in establishing specific semantic connections within learners’ prior cognitive schemas (Kaivanpanah and Rahimi, 2017; Elgort et al., 2018a; Butler, 2020). In contrast, another group of researchers advocates for internal-generated contextual clues created by EFL learners themselves, such as constructing sentences with new words. They emphasize the significance of generative semantic processing in improving vocabulary acquisition due to the variability of learners’ backgrounds and individual experiences (Sun and Scardamalia, 2010; Wittrock, 2010). Given that understanding provided contexts relies on learners’ prior language proficiency, example sentences may hinder semantic processing and contextual integration due to poor linguistic comprehensibility caused by the presence of unfamiliar words in the context (Bernardo and Harris, 2017; Chen et al., 2017; Elgort et al., 2018b). It further impedes learners’ vocabulary acquisition if example sentences are created by automatic machine translation that lacks the richness of expression (Hsiao and Hung, 2022). Learners might, therefore, achieve better performance by generating their contextual clues and linking words to their existing semantic networks (Ding et al., 2017).

Existing studies have highlighted the crucial function of contextual clues, whether provided by materials (e.g., example sentences) or generated by learners (e.g., creating sentences) in EFL vocabulary learning. However, few studies have explored the differences between these two approaches. Some evidence comes from incidental vocabulary learning, where learners memorize words incidentally through reading materials (Sok and Han, 2020). Learners were assigned to one of three tasks after reading a passage: multiple-choice, fill-in-the-blank, or sentence creation (Folse, 2006; Ansarin and Bayazidi, 2016). The results showed the poorest vocabulary retention when learning by creating sentences, indicating that contextual clues provided by materials enhance vocabulary acquisition more effectively than those generated by learners. However, the emphasis on additional word-related tasks and repetition in different contexts could have influenced the results in incidental vocabulary learning (Rott et al., 2002). The evidence from the aforementioned studies remains inadequate to conclusively establish the superiority of external contextual cues provided by content and materials. A contrasting study by Hulstijn and Laufer (2001) on incidental vocabulary acquisition revealed that learners who were instructed to write compositions using target words demonstrated superior vocabulary acquisition compared to those who engaged in a fill-in-the-blank task. This outcome underscores the efficacy of internal-generated contextual cues by learners.

Further evidence on this matter is derived from intentional vocabulary learning, where learners acquire new words by directly studying vocabulary lists (Sok and Han, 2020). Intentional learning is considered crucial in EFL vocabulary instruction and received much attention from researchers as it is the most commonly employed strategy among learners to acquire lexical knowledge (Yamamoto, 2014; Webb et al., 2020). There is an increasing consensus suggesting that intentional learning often results in better recall and retention performance compared to incidental learning (Schmitt, 2000; Yamamoto, 2014; Wong et al., 2021; Panmei, 2023). However, consensus remains elusive in the realm of intentional vocabulary learning. Some studies have suggested that both external-provided and internal-generated contextual clues have equal effects on promoting vocabulary acquisition (Talebzadeh and Bagheri, 2012; Soleimani et al., 2015). On the other hand, other researchers advocate the advantages of internal-generated contexts (Zhang, 2009; San-Mateo-Valdehita, 2023). San-Mateo-Valdehita (2023) observed that Japanese learners achieved better performance and reported greater cognitive efforts when learning Spanish vocabulary by creating their own sentences. However, this conclusion was drawn from a study on Spanish vocabulary, not EFL vocabulary learning. Zhang’s (2009) experiment yielded similar results, indicating that English major learners performed better when learning English vocabulary by constructing sentences rather than relying on example sentences provided by their instructor. Nevertheless, it is essential to acknowledge that most online EFL learners are non-majors who engage in informal self-directed vocabulary learning (Zourou, 2020; Pikhart et al., 2022). Their preferences for diverse language learning strategies stem from variations in vocabulary level and language proficiency compared to major learners (Shujing and Xie, 2007; Ma and Abdul Samat, 2022). Consequently, they might struggle with unknown words in provided contexts (Sadeghi and Nobakht, 2014). Moreover, the sentences they construct may not be as high in quality as those produced by major learners due to their limited vocabulary and knowledge of sentence structures (Nishida, 2014; Song et al., 2022). Overall, the debate regarding the benefits of contextual clues, whether provided by materials or generated by learners, warrants further exploration.

In addition to exploring behavioral performance, researchers argue that vocabulary acquisition can be predicted by learners’ mental efforts and cognitive involvement during the learning process (Zarifi et al., 2021). Current evidence suggests that vocabulary comprehension involves a deep level of processing linked to cognitive functions, which aid in retaining new words in long-term memory with a lasting impact (Craik and Lockhart, 1972; Craik and Tulving, 1975). Given the critical role of contexts in EFL vocabulary comprehension, it is important to investigate the differences in mental efforts between external-provided and internal-generated contextual clues. Research has shown that learners achieve better learning performance when context-related tasks require them to exert greater mental effort to understand word meanings (Verhallen and Bus, 2009), especially in intentional learning settings that demand higher attention and engagement with lexical knowledge (Zhang et al., 2020). In essence, the extent to which a new word is remembered depends on the level of cognitive involvement, particularly the mental efforts invested when encountering contextual clues (Keating, 2008; Taheri and Golandouz, 2021). However, prior studies have not reached a consensus on whether external-provided or internal-generated contexts necessitate higher cognitive involvement and elicit greater mental efforts from learners (Zou, 2017; Gohar et al., 2018; Alavinia and Rahimi, 2019; Liu and Reynolds, 2022). Soleimani et al. (2015) even suggested that learners appear to engage in similar mental efforts and achieve comparable performance when viewing presented contextual clues compared to generating their contexts, which may be attributed to the fact that EFL learners often prefer high-quality, easily understandable examples to grasp word meanings (Xu, 2006; Webb, 2008). Therefore, it remains imperative to further explore the differing effects of contextual clues provided by materials and those generated by learners from the perspective of mental efforts, especially for non-major EFL learners.

In addition, sustained attention has been identified as a critical predictor of online learning performance among learners (Chen and Wang, 2018). Focusing on lexical knowledge results in greater engagement in learning, which contributes to vocabulary acquisition (Ouyang et al., 2020). However, online learners often report difficulties in maintaining attention due to the lack of oversight and guidance (Valizadeh and Soltanpour, 2021). Attentional engagement in EFL vocabulary learning can be enhanced by increasing the frequency of exposure to words and task demands (Lai et al., 2017; Godfroid et al., 2018; Koval, 2019), which is distinct from the processes involved in viewing provided contexts and generating their contexts. Therefore, it is highly conceivable that learners may exhibit different levels of attentional engagement when acquiring lexical knowledge since they need to access contextual clues to understand unfamiliar words through various means. However, relevant studies have yet to explore this interesting and significant issue.

In addition to the limited exploration of learners’ mental efforts and attentional engagement during EFL vocabulary learning within the domain of contextual clues, another limitation of the existing literature is that researchers typically rely on behavioral self-reports after learning to assess cognitive activities. This limitation restricts the effectiveness of using semantic processing and contextual comprehension as predictors of vocabulary acquisition. Vocabulary comprehension is closely associated with deep cognitive functions and internal processing mechanisms (Crossley et al., 2009; Yousefi and Biria, 2018). However, behavioral measurements may not sufficiently capture learners’ cognitive activities, particularly their mental efforts during the learning process, as they are unable to reveal learners’ cognitive processes (Hulstijn, 1993; Yamada et al., 2014). Concerning attentional engagement, while several studies have explored learners’ visual preferences during online vocabulary learning using eye-tracking, these investigations were not directly related to the topic of contextual clues (Godfroid et al., 2018; Ouyang et al., 2020; Wang and Pellicer-Sanchez, 2022). Furthermore, eye movement indicators are associated with learners’ visual preferences but may not fully uncover their mental responses (Ding et al., 2022). Consequently, it is necessary to explore learners’ cognitive activities (such as mental efforts and attentional engagement) during vocabulary learning using an immediate and accurate method. This would contribute significantly to our understanding of the differences between the two ways of accessing contextual clues from a deeper and internal perspective.

### 1.3 Assessment of mental efforts and attentional engagement

It has been established that electroencephalography (EEG) can provide insights into the processes related to attention and mental efforts during learners’ cognitive activities, enabling a real-time examination through neural oscillations (Ko et al., 2017; Puma et al., 2018). Its reliability in assessing learners’ mental efforts has been established in an educational context (Zhu et al., 2021). Additionally, EEG has been employed to investigate online learners’ attentional engagement, given its sensitivity to variations in concentration (Chen et al., 2017; Chen and Wang, 2018). Therefore, EEG is a valuable tool for exploring learners’ mental efforts and attentional engagement when learning EFL vocabulary online using different approaches to accessing contextual clues. This approach helps address the research gaps and provides insights into the impact of contexts on vocabulary acquisition by examining learners’ neural activities.

Stronger beta-band oscillations (14–30 Hz) are reportedly associated with active cognitive involvement and sustained mental efforts (Sprengel and Job, 2004; Lin and Kao, 2018). This correlation is most prominent in frontal and parietal regions (Howells et al., 2010; Bauer et al., 2016; Orun and Akbulut, 2019) and is linked to learners’ self-control of cognitive processing and engaged mental efforts (McDonough et al., 2015; Stoll et al., 2016). Furthermore, an increase in alpha-band oscillations (8–13 Hz), especially in frontal and occipital regions, serves as an indicator of high cognitive loads when learners dedicate significant mental efforts to processing information (Meltzer et al., 2008; Wisniewski et al., 2017). Conversely, a decrease in alpha power is a sign of learners’ visual concentration when they focus on external target objects (Freunberger et al., 2011; Klimesch, 2012). It has been reported that the association between alpha power and attentional engagement is most pronounced in parietal and occipital regions (Jensen et al., 2002; Marsella et al., 2017; Whitmarsh et al., 2017), which are associated with learners’ processing and interpretation of visual information (Mazher et al., 2015). Another indicator related to attentional engagement is inter-subject correlation (ISC). ISC posits that there will be a greater degree of similarity in learners’ neural activities when they focus on the same visual stimulus (Cohen et al., 2017; Poulsen et al., 2017). In other words, EEG signals exhibit stronger correlations across learners when they attend to the same auditory or visual information than when their attention is directed to a mentally demanding task with high internal processing requirements (Ki et al., 2016). ISC helps overcome the subjectivity of self-reporting in behavioral measurement by investigating attentional engagement through the calculation of the correlation of neural oscillations among learners (Cohen et al., 2018).

### 1.4 The current study

As previously mentioned, contextual clues, whether provided by materials or generated by learners, have been the focus of related studies due to the pivotal role of contexts in EFL vocabulary acquisition. However, little emphasis has been placed on comparing the differing effects of provided and generated contextual clues on vocabulary learning. Furthermore, the mechanisms by which contexts influence learners’ EFL vocabulary learning processes remain unclear. Examining the neural underpinnings of cognitive activities, particularly mental efforts, and attentional engagement, during online vocabulary learning could enhance our understanding of how contextual clues are associated with vocabulary acquisition. Therefore, the present study compared two methods of accessing contextual clues (external-provided vs. internal-generated) and explores their effects on vocabulary learning among non-major EFL learners. Importantly, this study investigated the potential internal mechanisms, including mental efforts and attentional engagement, underlying these effects based on EEG technology.

The current study conducted a within-subject experiment in which two groups of vocabulary words were taught to participants through online video clips. Regarding external-provided contextual clues, participants were presented with example sentences to gain contextual clues for vocabulary comprehension after watching videos containing new words. For internal-generated clues, participants created their sentences to generate contextual clues. Learning performance was evaluated through post-tests, including scores and reaction times in key-press responses after learning. Given that internal-generated contextual clues tap into learners’ existing cognitive structures and may contribute to a better understanding of word meanings compared to provided contexts, the study formulated the following hypothesis regarding vocabulary acquisition:

Hypothesis 1: Learners will achieve better learning performance when they learn vocabulary words with contextual clues they generated themselves rather than those provided by materials.

In addition to behavioral indicators of learning performance, this study investigated learners’ cognitive activities through EEG measurements during vocabulary learning. First, it assessed their mental efforts during word comprehension and integration of contextual clues by examining alpha and beta-band oscillations. Second, the study investigated learners’ attentional engagement by analyzing alpha-band oscillations and ISC to explore the potential impact of different contextual clues on learners’ attention when acquiring lexical knowledge from videos. As self-generating contextual clues represent a more demanding task that may motivate learners to exert greater mental efforts and engage more in attention, the study formulated two hypotheses regarding cognitive activity:

Hypothesis 2: Learners will invest more substantial mental efforts, indicated by stronger alpha and beta-band oscillations when they comprehend vocabulary words with contextual clues generated by themselves compared to those provided by materials. Furthermore, stronger alpha-band oscillations will be most pronounced in frontal and occipital regions, whereas stronger beta-band oscillations will be most significant in frontal and parietal regions.

Hypothesis 3: Learners who learn vocabulary words with internal-generated contextual clues will demonstrate higher attentional engagement, as indicated by weaker alpha-band oscillation and higher ISC while viewing videos compared to those who learn with contextual clues provided by materials. Furthermore, weaker alpha-band oscillations and higher ISC will be most pronounced in the parietal and occipital regions.

## 2 Method

### 2.1 Participants

Twenty-nine non-major EFL students were recruited from a Chinese public university through an online advertisement, exhibiting female predominance (*n* = 21) with a mean age of 22.4 years (*SD* = 2.04), emanating from diverse academic backgrounds, including majors in educational technology, psychology, mechanical engineering, and others. All participants were native Mandarin Chinese speakers and reported having normal or corrected-to-normal vision and hearing. The learning material was high-frequency vocabulary words taken from preparation books for the Graduate Record Examination (GRE) (Pratheeba and Krashen, 2013). All participants should have passed College English Test-6 (CET-6) and had no prior preparation for GRE. CET-6 is the highest national English proficiency test for non-major students in China, and many undergraduates pass it with varying scores (Yang et al., 2013). This ensured a minimum level of English proficiency for participants to learn GRE words and complete the experimental tasks. Participants were not considered advanced English learners, as indicated by their pre-test scores (mean/maximum = 6.65/20, *SD* = 2.23). To provide context, advanced English learners in other studies typically scored an average of 105.29 out of 120 on the TOEFL (Moon et al., 2019). After the experiment, participants were compensated with 60 RMB as a token of appreciation. The study obtained informed consent from all participants and received approval from the Ethics Committee.

### 2.2 Design and procedure

The present study adopted a within-subjects design to control for the effects of prior knowledge. Each participant engaged in two experimental conditions categorized by the source of contextual clues: external-provided and internal-generated. In each condition, participants learned 40 vocabulary words through 40 video clips presented in random order. After viewing each video, participants were tasked with comprehending and remembering the word using contextual clues. For the external-provided contextual clue (condition a), an example sentence was displayed on the screen for 10 s without sound, and participants were instructed to read it silently. For the internal-generated contextual clue (condition b), participants had 10 s to create a sentence that included the word in mind. No specific requirements regarding sentence structure, content, or grammaticality were imposed. They then moved on to the next word in the following video clip. The order of the conditions was counterbalanced using a Latin Square design. Participant No.1 started with condition (a) and then proceeded to condition (b), while participant No. 2 followed the reverse order. The assignment of IDs was determined randomly based on the order of registration.

**Figure 1:**
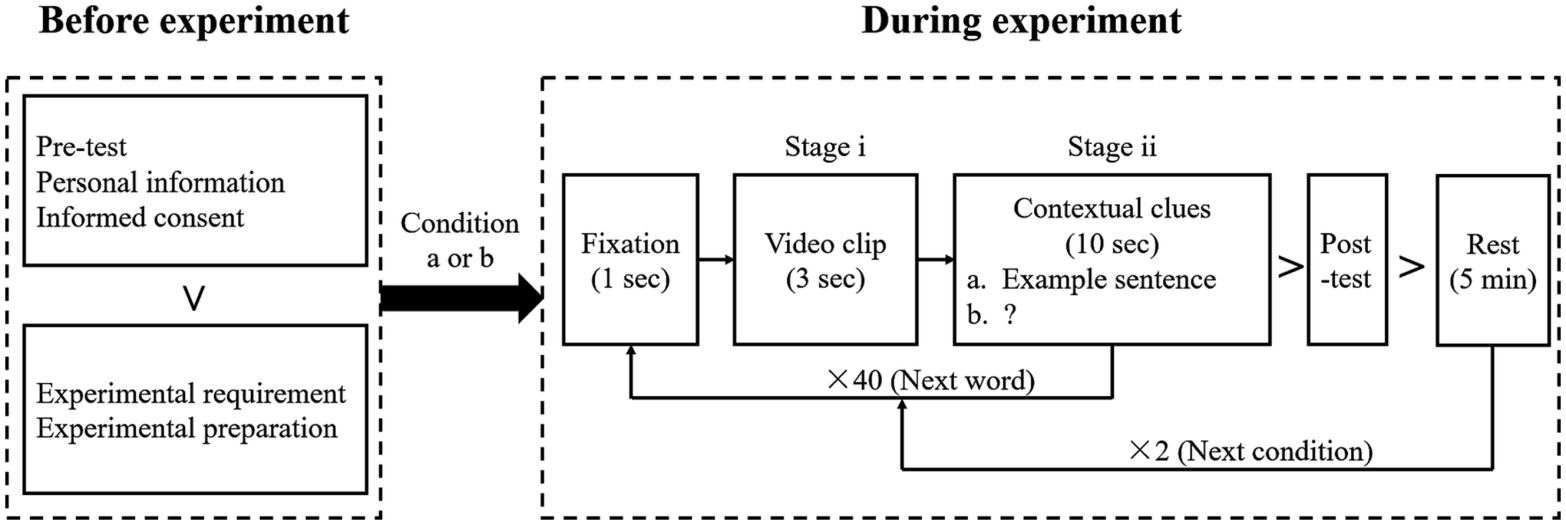
Experimental procedure. A flow diagram illustrating the things each participant did in experiment. The procedure in flow diagram is in line with the textual description in section 2.2 namely design and procedure.

Before the formal experiment, participants completed a pre-test to ensure that their English proficiency was not advanced, which might affect their vocabulary acquisition strategies and performance compared to non-advanced learners. Subsequently, they filled out a personal information questionnaire, provided informed consent, and were informed about the laboratory requirements before washing their hair and wearing an electrode cap. Following these preparations, participants engaged in both experimental conditions, with EEG signals recorded throughout the experiment. After learning 40 words in the first experimental condition, participants finished a post-test and took 5-min break to minimize carry-over and overload effects before enrolled in the other experimental condition. The entire experiment lasted approximately 1 h for each participant. The experimental procedure is depicted in Figure 1.

### 2.3 Material

Eighty words were randomly selected from the GRE vocabulary list, with each word taught through a three-second video clip. The English word and its Chinese translation were presented together on the left side of the screen. An instructor’s image appeared on the right side since it was proved to facilitate vocabulary learning from video lectures (Drijvers et al., 2019). She pronounced the word in English and provided its main meaning in Mandarin Chinese. The instructor did not use gestures, and her orientation and gaze remained consistent across all video clips to avoid the interference. Because these non-verbal behaviors are confirmed to act as social cues, which will influence learners’ attention and learning performance (Pi et al., 2020, 2021). The 80 video clips were randomly divided into two groups for use in the external-provided and internal-generated conditions (40 clips per group). There were no significant differences in video duration and the number of letters in words between the two groups [*t*(78) = 1.01, *p* = 0.317 > 0.05; *t*(78) = 1.13, *p* = 0.264 > 0.05]. To assess the difficulty of the two-group vocabulary words, 10 undergraduates from various majors (excluding English) watched the video clips and found that they were consistent in difficulty according to the result of an informal interview.

**Figure 2:**
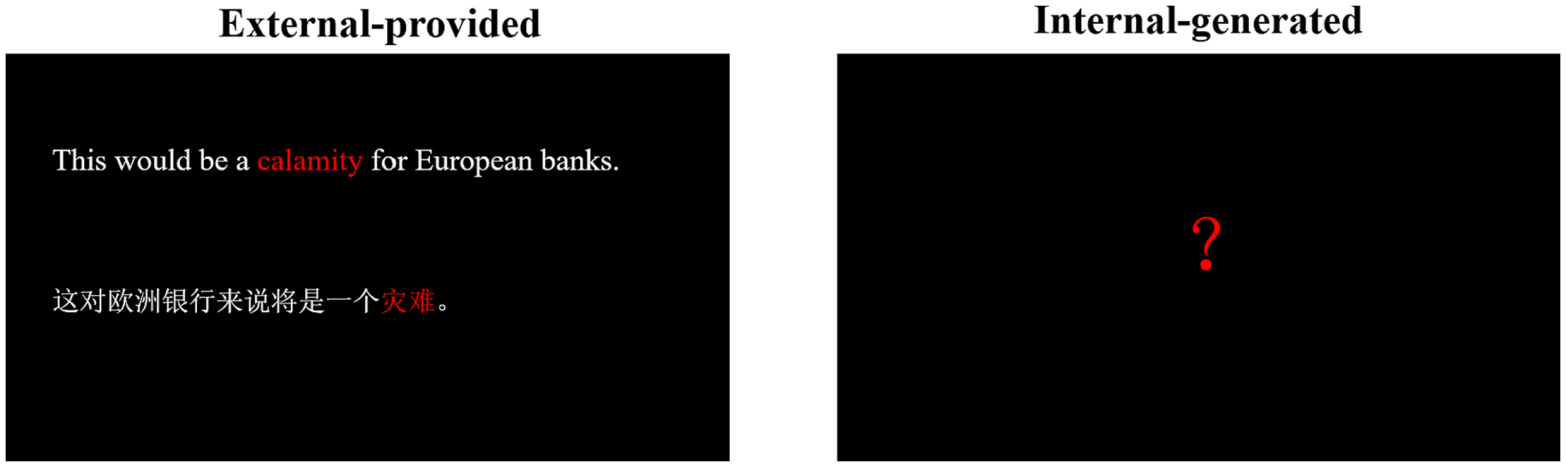
An example of contextual clues of vocabulary words. The left picture is for an example of contextual clues in the external-provided condition, presenting the context of word calamity. Texts on upper is an example sentence: This would be a calamity for European bank. Texts below is the translation of the example sentence in participants’ first language (Chinese): 这对欧洲银行来说将是一个灾难. The word calamity and its translation 灾难 are in red color while other words are in white. The right picture is for an example of contextual clues in the internal-generated condition, presenting a red? on screen.

Contextual clues for each word were presented following each video clip (Figure 2). For the external-provided contextual clue (condition a), example sentences for all words were sourced from online dictionaries (e.g., Youdao, Oxford, and Collins) by three English major postgraduates independently. These dictionary-derived example sentences were considered of high quality and suitable for facilitating vocabulary comprehension (Friedman, 2009; Liu and Mostow, 2013). Two English professors further reviewed and revised these sentences to ensure their appropriateness. Then, the 10 undergraduates selected the best example sentence for each word based on comprehensibility. The example sentences were accompanied by their Chinese translations as contextual clues, with the word and its meaning highlighted in red. For internal-generated contextual clue (condition b), participants were asked to create their sentences, and only a red “?” was displayed on the screen.

### 2.4 Measurements

#### 2.4.1 Prior knowledge (pre-test)

To assess learners’ prior knowledge of the learning content, a pre-test consisting of 20 multiple-choice questions was conducted. Each question corresponded to one word randomly selected from the pool of 80 words. Participants were required to choose the correct Chinese translation for the word from four options. Each correct answer earned participants 1 point, while incorrect answers received 0 points.

#### 2.4.2 Learning performance (post-test)

In each condition, 40 multiple-choice questions corresponding to the 40 words in that condition were used to assess participants’ mastery of vocabulary through key-press responses. The questions were developed by the two English professors. Participants were asked to choose the most suitable option to fill in the gapped text based on the compatibility between the word’s meaning and the context in the sentences. Each question included one correct option and three incorrect options, all derived from the 40 words within the respective condition. The frequency of occurrence for each word was balanced across questions. For example: *Question:* “*I pray that such a never comes again to anyone in the world*.” *Options: A. calamity; B. slap; C. magnify; D. queasy.* Participants received 1 point for each correct answer and 0 points for incorrect answers on each item, with a maximum score of 40 points in each condition. After choosing an option by pressing a key, the program automatically recorded reaction times and scores before proceeding to the next question. Both the external-provided and internal-generated contextual clues, post-tests exhibited high split-half discrimination [*t*(24) = 6.08, *p* < 0.001; *t*(24) = 5.46, *p* < 0.001].

#### 2.4.3 EEG recording and analysis

A 64-channel EEG electrode cap according to the international 10–20 system was placed on the surface of participant’s scalp to record EEG signals in conjunction with brain amplifier (Jasper, 1958). The electrode impedance was kept below 5 kΩ after inserting conductive gel into each electrode with a blunt needle syringe. CPz was respected to reference electrode during recording and the ground electrode was placed at the position of GND. The recording filtered with a passband from 0.1 to 100 Hz. There was no low-signal quality presented in EEG recording and no further data filtering or trimming was applied. EEG data analysis was performed using MATLAB. Bilateral mastoids M1 and M2 was acted as average re-reference in offline analysis to prevent laterality bias (Teplan, 2002). The original EEG signals were filtered with a passband between 0.1 and 50 Hz to remove the other artifact noises. Subsequently, the EOG and eye artifacts were eliminated by conducting independent component analysis (Mennes et al., 2010; Subasi and Gursoy, 2010). Algorithms were used in the software to flag and separate the epochs based on marks (Pi et al., 2021).

The pre-processed EEG data was re-segmented into specific time window as two stages: (i) learn words by videos (0–3 s) and (ii) comprehend words with contextual clues (3–13 s). The pre-video interval (−1 to 0 s) was acted as baseline correction. Short-time Fourier transform (STFT) was used to separate out the alpha-band (8–13 Hz) and beta-band (14–30 Hz) oscillations and compute their power (μV^2^) via averaging all scalp electrodes (Golden et al., 1973; Park et al., 2018; Krishnan et al., 2020). ISC was calculated based on covariance of within-subjects and between-subjects by integrating the feature vectors (Ki et al., 2016; Cohen et al., 2017). ISC and alpha-band oscillations in stage i were adopted to investigate the attentional engagement of participants when they learned words by videos, while alpha and beta-band oscillations in stage ii were used to explore their mental efforts when they comprehend words with contextual clues.

**Figure 3:**
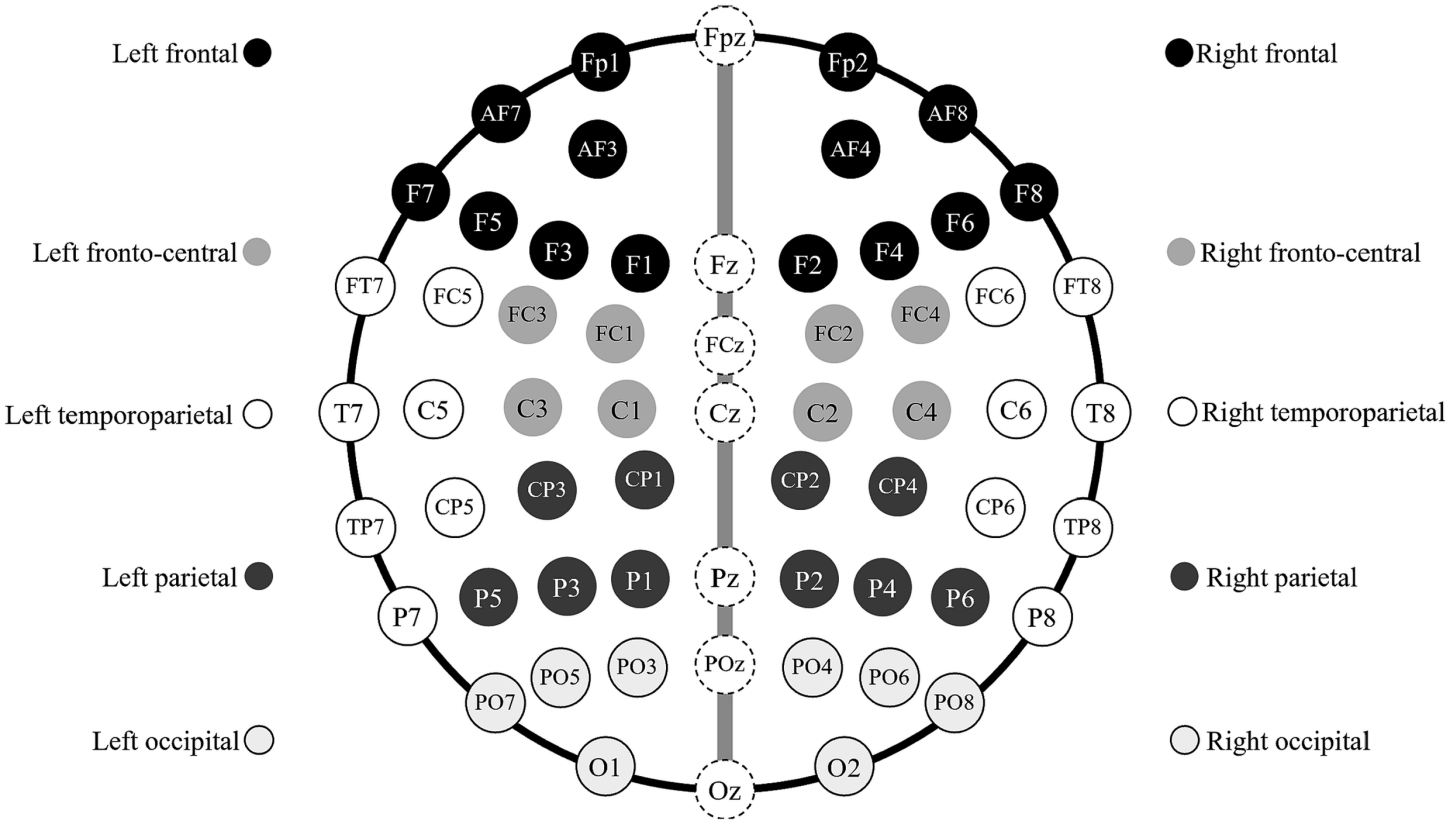
The electrodes in 10 brain regions. A picture illustrating the electrodes contained in each brain region. These regions and electrodes are consistent to textual description in the last paragraph of section 2.4.3 namely EEG recording and analysis.

EEG analysis was conducted to investigate the correlation between neural activities and brain regions. We clustered 61 electrodes (except CPz, M2 and M2 which used as reference) based on corresponding brain regions (Jahng et al., 2017), and further divided bilateral area according to midline (FPz, Fz, FCz, Cz, Pz, POz, Oz) to explore potential hemispheric dominance (Nobre et al., 2000). Following 10 regions showed in Figure 3 were used for analysis: (i) Left frontal (Fp1, AF3, AF7, F1, F3, F5, F7), (ii) Right frontal (Fp2, AF4, AF8, F2, F4, F6, F8), (iii) Left frontal-central (FC1, FC3, C1, C3), (iv) Right frontal-central (FC2, FC4, C2, C4), (v) Left parietal (CP1, CP3, P1, P3, P5), (vi) Right parietal (CP2, CP4, P2, P4, P6), (vii) Left occipital (PO3, PO5, PO7, O1), (viii) Right occipital (PO4, PO6, PO8, O2), (ix) Left temporoparietal (FT7, FC5, T7, C5, TP7, CP5, P7), and (x) Right temporoparietal (FT8, FC6, T8, C6, TP8, CP6, P8).

## 3 Results

### 3.1 Learning performance

To assess differences in learning performance between the two types of contextual clues, paired samples *t*-tests in SPSS 22.0 were conducted with scores and reaction times as the dependent variables. The data were tested using the Shapiro–Wilk normality test and met the normality assumption of the *t*-test (Pallant, 2016). The “Condition” (external-provided vs. internal-generated) as the within-subjects independent variable. Cohen’s *d* (small size: 0.2–0.5, medium size: 0.5–0.8; large size: > = 0.8) was used to measure effect size for the *t*-tests according to Cohen (1988).

The results revealed significant differences in learning performance between the two types of contextual clues (Table 1). With internal-generated contextual clues, participants achieved higher scores [*MD* = 3.42, *t*(25) = 3.72, *p* = 0.001 < 0.05, *d* = 0.73] and shorter reaction times [*MD* = –1.32, *t*(24) = −2.06, *p* = 0.05, *d* = 0.40] compared to the external-provided contextual clues. These results strongly support Hypothesis 1, which suggests that participants benefit more from generating their own contextual clues than reading contextual clues provided by learning materials for vocabulary learning.

**Table 1:** Mean and standard deviation of learning performance in two conditions.

Condition	Scores		Reaction time (s)	
	*M*	*SD*	*M*	*SD*
External-provided	20.35	6.20	16.82	5.14
Internal-generated	23.77	5.38	15.50	3.79

### 3.2 EEG evidence

To investigate differences in participants’ mental efforts and attentional engagement between the two types of contextual clues, repeated-measures ANOVAs (2 × 10) in SPSS 22.0 were performed on EEG oscillations (alpha and beta-band) and ISC as the dependent variables, with “Condition” (external-provided vs. internal-generated) and “Region” (Left frontal, Right frontal, Left fronto-central, Right fronto-central, Left parietal, Right parietal, Left occipital, Right occipital, Left temporoparietal, Right temporoparietal) as within-subject independent variables. Mauchly’s test was employed to assess the assumption of sphericity for repeated-measures analysis (Pallant, 2016). In case the assumption of sphericity was violated, Greenhouse–Geisser corrected significance values were reported. Effect sizes were measured using *η*^2^ (small size: 0.01–0.06; medium size: 0.06–0.14; large size: > = 0.14) for the ANOVAs (Cohen, 1988).

#### 3.2.1 Attentional engagement during learning words from videos

##### 3.2.1.1 Alpha-band oscillations

**Figure 4:**
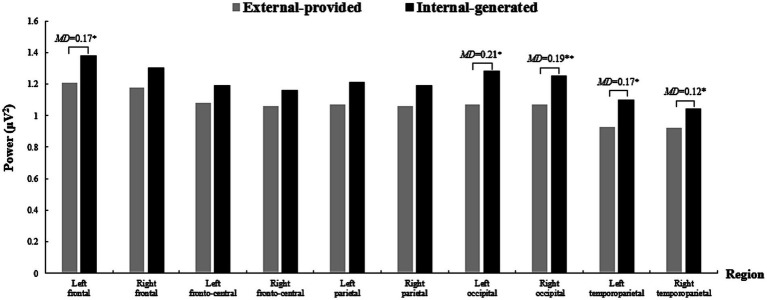
The power of alpha-band oscillations across brain regions in two conditions when learning words by videos. *p < 0.05, **p < 0.01, ***p < 0.001; MD is the mean difference between two Conditions (the same below). A 10 section bar graph plotting the power of alpha-band oscillations of participants when they learned words by videos. Each section responding to a brain region, from left to right namely Left frontal, Right frontal, Left fronto-central, Right fronto-central, Left parietal, Right parietal, Left occipital, Right occipital, Left temporoparietal and Right temporoparietal (the same below). Each section contains two bars, the gray one on the left represents the external-provided condition, whereas the black one on the right represents the internal-generated condition (the same below). Significantly lower alpha power occurs in the external-provided condition in Left-frontal, Left occipital, Right occipital, Left temporoparietal and Right temporoparietal regions indicated by obvious shorter gray bar on the left than black bar on the right in first, seventh, eighth, nineth, and tenth sections.

Significant main effects were observed for “Condition” [*F*(1, 25) = 4.87, *p* = 0.04 < 0.05, *η*^2^ = 0.16) and “Region” (*F*(9, 225) = 11.85, *p* < 0.001, *η*^2^ = 0.32] regarding alpha-band oscillations. More importantly, the interaction effect of “Condition × Region” was statistically significant [*F*(9, 225) = 1.97, *p* = 0.04 < 0.05, *η*^2^ = 0.07]. Simple effect analysis indicated that with external-provided contextual clues, participants exhibited weaker alpha power in the left frontal, bilateral occipital, and temporoparietal regions (Figure 4). However, differences in alpha power were not pronounced in the parietal region as expected.

##### 3.2.1.2 Inter-subject correlation

**Figure 5:**
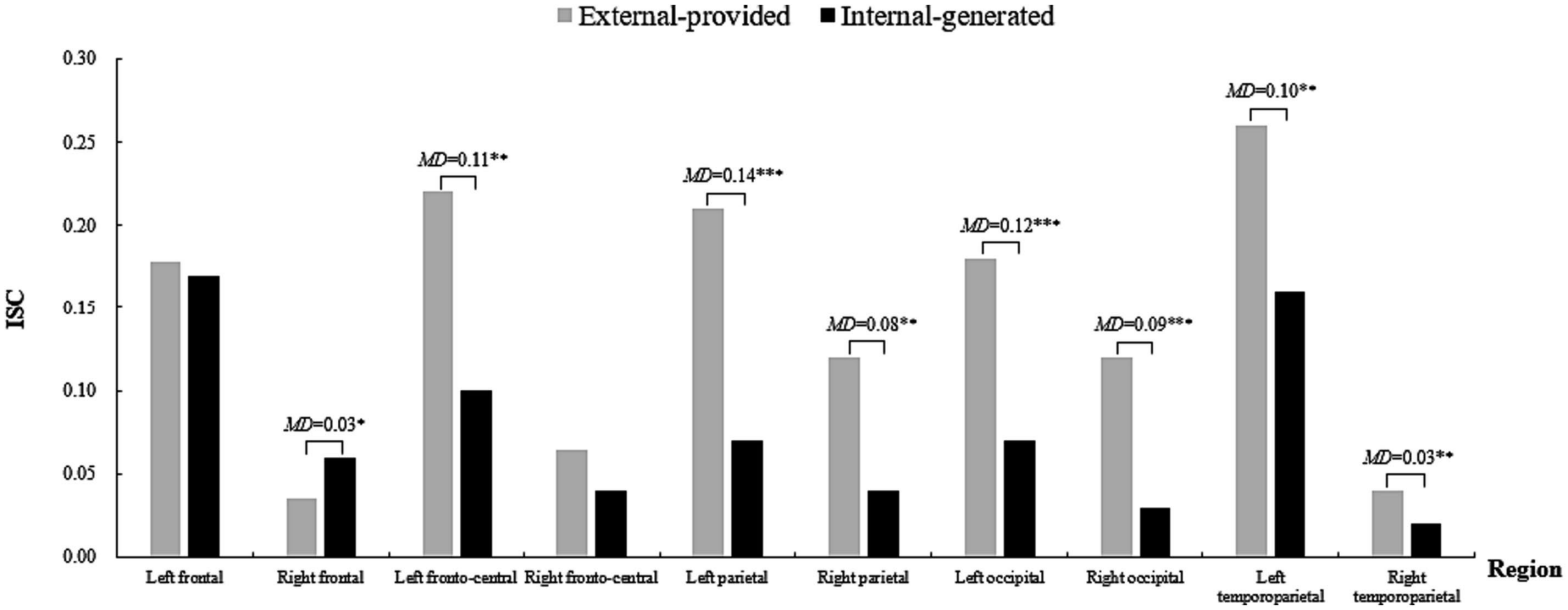
The ISC across brain regions in two conditions when learning words by videos. A 10 section bar graph plotting the ISC of participants when they learned words by videos. Significantly higher ISC occurs in the external-provided condition in Left-fronto-central, Left parietal, Right parietal, Left occipital, Right occipital, Left temporoparietal and Right temporoparietal regions, indicated by obvious higher left gray bar than right black bar in third, fifth, sixth, seventh, eighth, nineth, and tenth sections. However, a higher black bar on the right than gray bar on the left in second section indicates significantly lower Left-frontal ISC in the external-provided condition.

Significant main effects were observed for “Condition” [*F*(1, 25) = 15.49, *p =* 0.001 < 0.05, *η*^2^ = 0.38] and “Region” [*F*(9, 215) = 68.38, *p < 0*.001, *η*^2^ = 0.73] in relation to ISC. Additionally, the “Condition × Region” interaction was significant [*F*(9, 225) = 11.67, *p* < 0.001, *η*^2^ = 0.32]. Simple effect analysis indicated that with external-provided contextual clues, higher ISC was observed, especially in the left fronto-central, bilateral parietal, occipital, and temporoparietal regions. Interestingly, higher right frontal ISC was found in the internal-generated condition (Figure 5).

In summary, these results were inconsistent with Hypothesis 3, which posited that contextual clues would influence participants’ attentional engagement during video viewing. Weaker alpha-band oscillations and higher ISC were observed with external-provided contextual clues rather than with internal-generated contextual clues. Moreover, differences in alpha-band oscillations were significant in the left fronto-central, bilateral occipital, and temporoparietal regions but not in the parietal region as anticipated. ISC differences were observed in left fronto-central and bilateral temporoparietal regions other than the expected parietal and occipital regions, with a contrasting ISC discrepancy found in the right frontal region.

#### 3.2.2 Mental efforts during comprehending words with contextual clues

##### 3.2.2.1 Alpha-band oscillations

**Figure 6:**
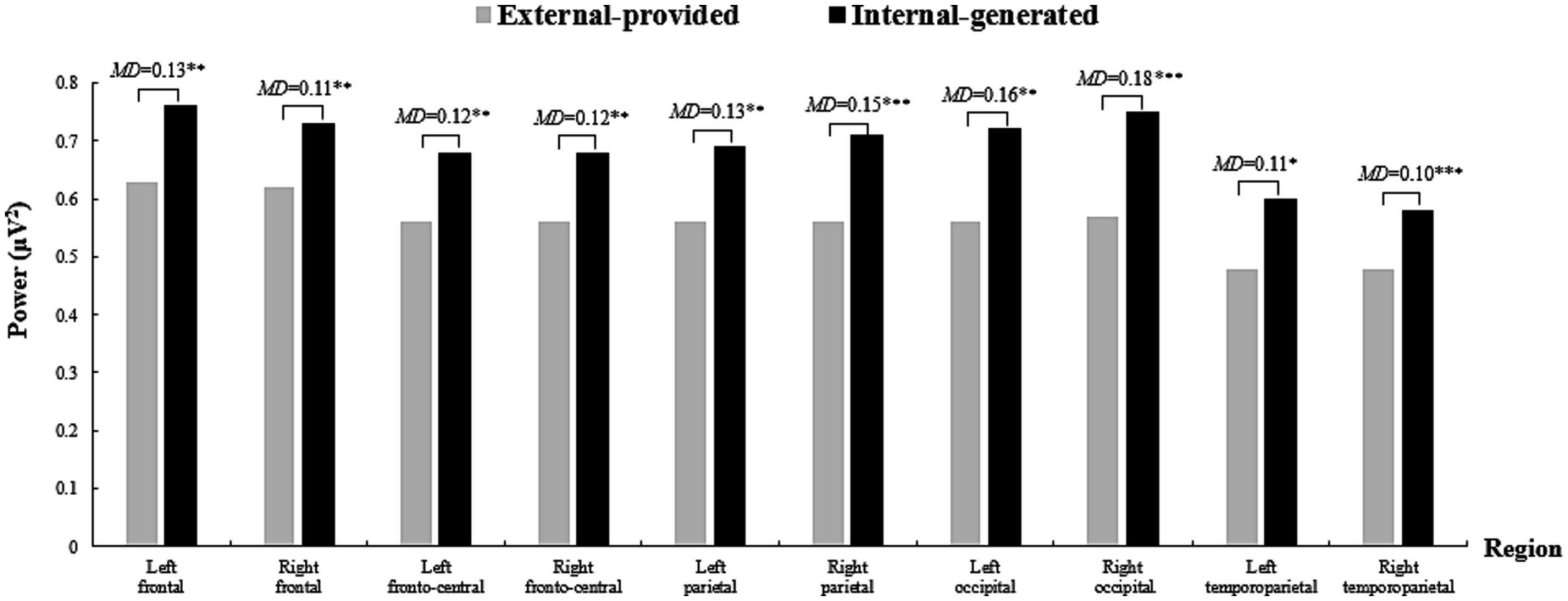
The power of alpha-band oscillations across brain regions in two conditions when comprehending words with contextual clues. A 10 section bar graph plotting the power of alpha-band oscillations of participants when they comprehend words with contextual clues. Significantly higher alpha power occurs in the internal-generated condition in all brain regions indicated by obvious higher black bar on the right than gray bar on the left in each section.

Significant main effects were observed for the factors “Condition” [*F*(1, 25) = 15.49, *p* = 0.001 < 0.05, *η*^2^ = 0.38] and “Region” [*F*(9, 225) = 13.14, *p* < 0.001, *η*^2^ = 0.35] in relation to alpha-band oscillations. Importantly, the interaction effect of “Condition × Region” was statistically significant [*F*(9, 225) = 2.93, *p* = 0.043 < 0.05, *η*^2^ = 0.11]. Simple effect analysis was conducted, revealing that with internal-generated contextual clues, participants exhibited higher alpha power in bilateral frontal, fronto-central, parietal, occipital, and temporoparietal regions (Figure 6).

##### 3.2.2.2 Beta-band oscillations

**Figure 7:**
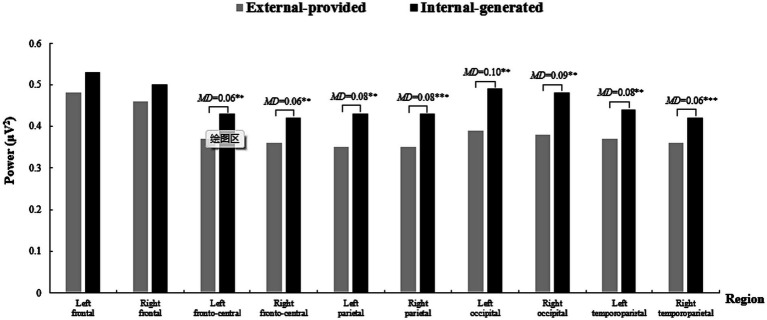
The power of beta-band oscillations across brain regions in two conditions when comprehending words with contextual clues. A 10 section bar graph plotting the power of beta-band oscillations of participants when they comprehend words with contextual clues. Significantly higher beta power occurs in the internal-generated condition in all brain regions except Left and Right frontal indicated by obvious higher black bar on the right than the gray bar on the left from third to tenth sections.

Significant main effects were observed for the factors “Condition” [*F*(1, 25) = 12.22, *p* = 0.002 < 0.05, *η*^2^ = 0.33] and “Region” [*F*(9, 225) = 15.98, *p* < 0.001, *η*^2^ = 0.39] in relation to beta-band oscillations. Additionally, the interaction effect of “Condition × Region” was statistically significant [*F*(9, 225) = 4.68, *p = 0*.006 < 0.05, *η*^2^ = 0.16]. Simple effect analysis showed that with internal-generated contextual clues, participants exhibited higher beta power in bilateral fronto-central, parietal, occipital, and temporoparietal regions (Figure 7). However, a significant difference in beta power was not observed in the frontal region.

Taken together, our results partially supported Hypothesis 2, indicating that with internal-generated contextual clues, participants exerted greater mental efforts, as indicated by stronger alpha and beta-band oscillations when comprehending vocabulary words compared to the external-provided contextual clues. However, the differences in alpha-band oscillations extended across the entire brain except the frontal and parietal regions. Besides, the differences in beta-band oscillations were observed in other regions but not in the frontal region as expected.

## 4 Discussion

The current study aimed to evaluate the impact of contextual clues provided by materials versus those generated by learners on online EFL vocabulary acquisition. Our findings suggested that internal-generated contextual clues brought more significant benefits for vocabulary acquisition compared to contexts provided by materials. Learners achieved higher learning performance, as indicated by better scores and shorter reaction times. EEG signals indicated increased mental efforts during word comprehension but reduced attentional engagement when viewing lexical knowledge from videos. To our knowledge, no study has hitherto compared the effects of contextual clues between external-provided and internal-generated sources on online vocabulary learning, especially for non-major EFL learners. Moreover, it is the first to adopt EEG to provide underlying neural evidence for the benefits of contextual clues from the perspectives of mental effort and attentional engagement.

### 4.1 The impact of contextual clues on EFL vocabulary learning performance

The learning performance in vocabulary comprehension significantly differed between the external-provided and internal-generated contextual clues. Learners achieved higher scores and shorter reaction times when learning new words with self-created contexts. These results align with Zhang’s (2009) findings, suggesting that sentence creation leads to better vocabulary acquisition than viewing example sentences, even for non-major EFL learners in this study. According to constructivist learning theory, internal-generated contextual clues are derived from learners’ cognitive structures associated with their prior experiences and knowledge (Chuang, 2021). This process enables learners to construct understandable semantic associations between new words and existing lexical resources, resulting in accurate subsequent retrieval (Crutcher and Ericsson, 2003). This advantage was further supported by the reaction times in the post-test, where learners were asked to recall word meanings. An increase in reaction times reflects higher task demands associated with more challenging recall and retrieval (Bachurina et al., 2022). Shorter reaction times associated with internal-generated contextual clues indicated efficient retrieval performance during recall, suggesting that learners had solidified semantic connections between unfamiliar words and existing schema after generating contextual clues for them (Cook and Ausubel, 1970). However, these results contrast with several studies that advocate for equal benefits of generated and provided contextual clues (Talebzadeh and Bagheri, 2012; Soleimani et al., 2015), with some incidental vocabulary learning studies even presenting empirical evidence for the superior advantages of contexts presented by content and materials (Folse, 2006; Ansarin and Bayazidi, 2016). This inconsistency might be attributed to heterogeneity across studies in terms of the externally provided contextual clues. Importantly, the present study limited the richness and quantity of contexts to one example sentence for each word, whereas in previous studies, researchers provided a paragraph or more than one sentence, offering abundant contextual clues. This limitation might have mitigated evidence of the expected benefits of provided contexts, given that a single example might not be sufficient for learners to fully grasp word meanings and usage (Frankenberg-Garcia, 2012). Indeed, example sentences can better leverage their unique advantages in vocabulary comprehension by providing various contextual clues for each word, particularly for words with multiple implications in different situations (Han and Song, 2011; Huang et al., 2019). However, learners may struggle to find multiple examples as online English vocabulary learning instruments usually contain no more than one example sentence used for describing the context and explaining word meaning (Huang and Ku, 2016; Wang et al., 2021). Nevertheless, these results presented the first evidence of the superior benefits of internal-generated contextual clues on vocabulary acquisition compared to single-exposure contexts provided by materials.

### 4.2 The impact of contextual clues on EFL learners’ mental efforts during words comprehension

The improved learning performance associated with the internal-generated contextual clues also stemmed from learners’ significantly higher mental efforts during word comprehension compared to the external-provided condition. This study assessed learners’ mental efforts using EEG frequencies in the alpha and beta-bands, which are associated with learners’ cognitive activities (Chikhi et al., 2022). Learners’ alpha-band oscillations are stronger when they actively control their cognitive resources to handle tasks (Zoefel et al., 2011; Huycke et al., 2021). Increased alpha activities in the frontal and occipital regions represent the maintenance of mental efforts and a high level of working memory load, leading to subsequent cognitive fatigue (Meltzer et al., 2008; Wascher et al., 2014). Higher beta power, especially in the frontal and parietal regions, also reflects significant cognitive involvement in the current task when solving problems (Tschentscher and Hauk, 2016; Hubner et al., 2018). For example, learners’ beta power increases as they devote their best mental efforts to satisfying high task demands and achieving better task performance (She et al., 2012). Greater mental efforts thus contribute to positive information processing in working memory (Jaquess et al., 2018; Zhu et al., 2021), which facilitates deeper semantic integration and vocabulary comprehension (Ender, 2016; Bohn et al., 2021).

Significant stronger oscillations in the alpha and beta bands were associated with internal-generated contextual clues compared to external-provided clues. The results indicated that learners devoted greater mental efforts to comprehending words when they self-created contextual clues. Given that the generative task, as a transfer of knowledge, enhances learners’ autonomy and motivation in vocabulary learning (Laufer, 2001; Jilani and Yasmin, 2016), it is associated with mental efforts and the cognitive resources invested (Seufert, 2020). However, in the present study, a difference in alpha power was observed in all regions except the frontal and occipital regions that were expected (i.e., bilateral fronto-central, parietal, and temporoparietal regions). In contrast, beta power differed significantly between conditions in bilateral fronto-central, parietal, occipital, and temporoparietal regions, except the frontal region that was assumed. These results indicated the whole-brain effects of semantic processing when learners comprehended words with contextual clues in EFL vocabulary learning.

On the one hand, stronger alpha-band oscillations were observed in all regions with internal-generated contextual clues compared to external-provided contextual clues. Greater alpha power in the frontal and occipital regions revealed that learners devoted greater efforts to encoding word meanings, reflecting high demands for cognitive resources during internal processing (Meltzer et al., 2008; Benedek et al., 2011; Wascher et al., 2014). We also found higher alpha power in the fronto-central, parietal, and temporoparietal regions, where is relevant to semantic processes. The activation of the frontal and temporoparietal regions indicates successful semantic integration after learners match words with related contextual clues (Baumgaertner et al., 2002; Rempe et al., 2022), whereas neural activity in the fronto-central results from functional coupling with the frontal region when increased executive control of semantic processing occurs (Rominger et al., 2020). It has been established that the process of semantic integration involves the retrieval of prior contextual clues in learners’ long-term memory, resulting in alpha excitation associated with good semantic integration in the regions mentioned above (Ehrhardt et al., 2022). In addition, increasing alpha power in frontal, fronto-central, occipital, and parietal regions is also related to creative idea generation and thinking activities instead of resting states (Rominger et al., 2018; Barcelona et al., 2020; Rominger et al., 2022). This demonstrates learners’ creative thinking and original ideas when they internal-generate contextual clues, enabling them to devote greater mental efforts compared to acquiring knowledge generated by others (Wen, 2020). In short, the increased alpha-band oscillations in the above regions suggest better cognitive task performance, which requires higher working memory demands (Mahjoory et al., 2019). The process through which learners understand vocabulary words with internal-generated contextual cues is characterized by effective internal processing, substantial cognitive exertion, and seamless semantic integration.

On the other hand, stronger beta-band oscillations were observed in all regions except the frontal region with internal-generated contextual clues compared to the external-provided contextual clues. The activation of frontal beta activity is associated with executive function and cognitive control resulting from learners’ active engagement in current cognitive tasks (Kropotov, 2009; Basharpoor et al., 2019). In other words, increased beta power in the frontal region represents a state of efficient cognitive functioning (Song et al., 2014). The comparable beta power in the frontal region between conditions demonstrated that learners had involved similar cognitive resources in vocabulary encoding and comprehension, whether with generated or provided contextual clues. However, higher parietal beta power was associated with internal-generated contextual clues since learners’ recall processes typically involve retrieving contextual clues from their prior knowledge, alongside cognitive involvement during semantic processing (Tschentscher and Hauk, 2016; Kaiser et al., 2017). Besides the expected regions, greater beta power was also observed in the fronto-central, occipital, and temporoparietal regions when learners generated contexts themselves. Higher beta power found in the fronto-central region was consistent with several language learning studies, suggesting learners’ cognitive involvement and active processing (Subbaraj et al., 2014; Alimardani et al., 2021). Moreover, greater beta power in the occipital and temporoparietal regions has been associated with learners’ perception of difficulty and increased tension due to high task demands, which often predict better task performance (Kakizaki, 1985; She et al., 2012). The above studies overlap in their assertion that the process of semantic integration and processing is similar to language grammar learning, driven by whole-brain functional connectivity communicated through beta-band oscillations (Kepinska et al., 2017). The interregional communication of brain activation from anterior to posterior is modulated by working memory demands, which is associated with learners’ mental efforts and cognitive involvement (Sauseng et al., 2005; Fernandez et al., 2021). In brief, learners devote higher mental efforts to being involved in the process of contextual retrieval and integration when they comprehend vocabulary words with internal-generated contextual clues due to the high demands for cognitive resources from tasks.

### 4.3 The impact of contextual clues on EFL learners’ attentional engagement in videos of lexical knowledge

This study further investigated learners’ attentional engagement when learning lexical knowledge from videos using alpha-band oscillations and ISC. A decrease in alpha-band oscillations in parietal and occipital regions indicates active attention to external visual information (Sokoliuk et al., 2019; Son et al., 2023), while higher ISC caused by similar neural activities reveals learners’ better attentional engagement when processing the same visual stimuli (Cohen et al., 2017, 2018). A high attention level suggests that learners are focusing on and processing presented visual information, contributing to its subsequent encoding and retrieval in memory (Kirkorian et al., 2016; Kruikemeier et al., 2018). As a result, learners achieve a high level of learning performance, marked by positive attentional engagement, especially in online self-directed environments (Chen et al., 2017; Wang et al., 2019).

Interestingly, we found that learners showed significantly lower attentional engagement during viewing videos when learning with internal-generated contextual clues compared to learning with the external-provided contextual clues, based on the alpha power and ISC. In this respect, pronounced higher alpha power was associated with internal-generated contextual clues, especially in the left frontal, bilateral occipital, and temporoparietal regions, except for the parietal region we anticipated. Alpha power in the parietal region is associated with visual attention to external stimuli (Hutchinson et al., 2021). The comparable parietal alpha power between the two conditions showed that learners paid attention to videos and actively received lexical knowledge. Besides, the left frontal region plays a significant role in language processing (Plaza et al., 2009), and stronger frontal alpha activity reflects learners’ internal processing, where they proactively inhibit unrelated visual stimuli and focus on key details (Benedek et al., 2011). Higher alpha power in the left frontal region demonstrated that learners engaged in a proactive process of eliminating irrelevant visual information from videos and then focused on the internal processing of crucial lexical knowledge. Stronger alpha power was also pronounced in the occipital region, indicating learners’ inhibition of distracting visual input and the reallocation of sensory resources (van Diepen and Mazaheri, 2017). In addition, excited temporoparietal alpha is associated with efficient visual search resulting from perceptual prediction (Spaak et al., 2016), which suggests that learners can predict the spatial location of key knowledge in upcoming video clips due to its design consistency. These results suggest an interesting finding that when learners comprehend words with internal-generated contextual clues, learners actively selected important content for subsequent processing and filtered unrelated visual input via top-down attentional control mechanisms when learning lexical knowledge through videos. The active attentional control may be due to the generative task of self-creating contextual clues, which improves learners’ goal-driven attention and results in prioritized processing of task-related information in working memory (Ravizza et al., 2021), suggesting that self-generating contextual clues might further play an essential function in subsequent visual tasks, which is similar to active cognitive control for reducing interference from distractors and maintaining priority attention to core concepts (Lavie et al., 2004).

Secondly, the present study also found higher ISC was associated with external-provided contextual clues rather than internal-generated contextual clues, especially in the right frontal, left fronto-central, bilateral parietal, occipital, and temporoparietal regions. The activation of the parietal and occipital regions reflects learners’ processing of visual information (Mazher et al., 2015). A stronger ISC in these regions indicates that learners viewed the visual information with similar psychological perspectives and shared understanding (Lahnakoski et al., 2014). Additionally, a greater ISC was found in the temporoparietal region, which is involved in bottom-up visual selection directed by stimuli (Corbetta and Shulman, 2002) and is associated with the initial recognition and acquisition of vocabulary words (Mills et al., 1993; Davis and Gaskell, 2009). These results demonstrate that learners in the external-provided condition exhibited higher consistency in recognizing and visually processing lexical knowledge, with their attention directed by the stimuli in the videos. Moreover, the fronto-central and temporoparietal regions are associated with language processing in phonological tasks (Seghier et al., 2004; De Carli et al., 2007). The left fronto-central region is designated for the auditory pre-attentive processing of word perception (Arunphalungsanti and Pichitpornchai, 2018), whereas the temporoparietal region plays an essential role in auditory comprehension by transforming auditory input into mental lexical representation (Bosseler et al., 2021). The higher ISC in these regions appears to indicate more significant auditory participation in attending to and recognizing lexical knowledge for learners when they learn with the external-provided contextual clues. In summary, when learners need to comprehend word meanings by viewing provided contextual clues, they focus on the learning content in videos and exhibit higher sensory engagement in lexical knowledge. The greater focus on external input information likely results from the receptive task of viewing presented contexts, promoting learners to attach great importance to all lexical knowledge from videos through a bottom-up mechanism (Kakvand et al., 2022). Intriguingly, in the present study, learners with higher attentional engagement to instructional videos yielded worse vocabulary acquisition compared to those whose attention to videos seems lower. According to the uniquely higher ISC in the right frontal region in the internal-generated condition, we assumed that it might result from learners’ earlier initial processing of lexical knowledge after recognizing unfamiliar words from videos. This finding aligns with prior language learning studies, which suggested a positive correlation between right frontal engagement and better acquisition and retention when learners preliminarily processing language knowledge (Qi et al., 2019). This indicates that mind wandering of attention during learning through videos is not always detrimental; some off-tasks thinking about topics can enhance knowledge acquisition (Kane et al., 2017). The results suggest an intriguing finding that greater attentional engagement might not necessarily predict better learning performance from video lectures, especially in online vocabulary learning. Instructional information with low complexity, such as lexical knowledge, might not require learners’ excessive attentional resources to the learning content. Instead, learners’ active processing after recognizing the key knowledge is the most critical factor.

In summary, the mixed results of this study suggest that self-generating contextual clues to understand word meanings motivate learners to exert greater mental efforts in semantic processing and lead to better contextual integration. This generative task further leads to a top-down attentional mechanism when learners learn lexical knowledge from videos and enables them to process important visual information as a priority. The active control of the cognition process by EFL learners consequently improves their online vocabulary learning performance.

## 5 Significance and implications

The present study has improved the current understanding of the influence of two ways of accessing contextual clues on online EFL vocabulary learning. Existing studies have primarily focused on whether presenting contexts along with words facilitates vocabulary acquisition (Bilgin and Tokel, 2019; Nielsen et al., 2022), with different means of accessing contextual clues largely understudied (Zhang, 2009; San-Mateo-Valdehita, 2023). This study compared two contextual clues, referring to their source: those provided by materials and those generated by learners. The results were drawn based on both behavioral and neural evidence, confirming the superiority of learning vocabulary with internal-generated contexts compared to external-provided contexts.

Importantly, our findings about contextual clues have meaningful implications for online vocabulary learning. Firstly, learners who generate contextual clues themselves appear to devote greater mental efforts to semantic processing and word comprehension compared to those who receive provided contextual clues. This contributes to better vocabulary learning performance, even with higher demands for cognitive resources. Learners are encouraged to self-create contextual clues for vocabulary words after recognizing them to achieve satisfactory acquisition. Secondly, self-generating contextual clues further enhance top-down attentional control and promote the priority processing of crucial visual input when learners view lexical knowledge through videos. Therefore, learners should pay selective attention to the presented learning content from online materials and actively process the most important knowledge.

Our results also provide further implications for the design of online EFL vocabulary instructional instruments. On the one hand, compared to presenting contextual clues for each word, requiring learners to self-create contexts motivates their higher mental efforts and cognitive involvement. The greater engagement subsequently facilitates learners’ better vocabulary acquisition and may further improve their persistence in autonomous online learning. For another, lexical knowledge in online materials should be re-considered and simplified to help learners gain crucial information at first sight. The refined content consequently frees learners from the distraction of unimportant input and facilitates optimal use of visual resources to preferentially process crucial information.

Importantly, this study is the first to explore the neural underpinnings of learners as they engage in the comprehension of words using contextual clues provided by materials or internal-generated for semantic processing and contextual integration. We adopted EEG oscillations to explore learners’ mental efforts, while previous studies about context investigated semantic comprehension by event-related potentials (ERPs), especially N400 (Abel et al., 2018; Bell et al., 2019). Specifically, higher alpha and beta-band oscillations were associated with the internal-generated contextual clues compared to the external-provided contextual clues, indicating learners’ greater mental efforts and cognitive involvement in semantic processing and the results of better semantic integration (Zoefel et al., 2011; Tschentscher and Hauk, 2016; Hubner et al., 2018; Huycke et al., 2021). Moreover, the present study further investigated learners’ attentional engagement in lexical knowledge from videos influenced by different ways of accessing contextual clues, which has never been considered in other studies on this topic. The outcomes of higher alpha power and lower ISC with internal-generated contextual clues compared to the external-provided contextual clues revealed learners’ goal-directed attentional control and their selective visual processing of critical information when viewing videos (Cohen et al., 2017, 2018; Sokoliuk et al., 2019; Son et al., 2023). These results collectively suggest that EFL learners who generate contextual clues themselves engage more cognitive resources in semantic processing and word comprehension, whereas their attentional engagement in lexical knowledge when viewing videos remains relatively low, which mainly stems from learners’ autonomous cognitive processes containing a top-down attentional mechanism and active information processing, enabling them to attend to critical incoming information of lexical knowledge as a priority and then integrate new words with related contexts in prior knowledge structures with mental efforts. These findings provide a further explanation of the different effects between provided and generated contextual clues on online EFL vocabulary learning, as well as extending previous behavioral studies on this topic (Zhang, 2009; San-Mateo-Valdehita, 2023) by clarifying learners’ cognitive activities in terms of attentional engagement and mental efforts.

## 6 Limitations and further work

There are three limitations in this study that can be addressed in future research. First of all, we did not require participants to write down the sentences they created or type them on the screen. Even though we had reminded participants before the experiment to create sentences in English as contextual clues, we cannot actually control the language they are thinking in. The sentence-making in Mandarin Chinese in their mind would potentially influence the effect of contextual clues on vocabulary acquisition. Because generative tasks facilitate learners to understand and use vocabulary words through a process of exposure and contextual integration that nurtures their language proficiency (Ha and Bellot, 2020). In addition, the lack of recorded sentences against further analysis of their structure, content, and grammaticality. Given that this was a preliminary trial undertaken to explore the different effects of provided and generated contextual clues, we aimed to investigate learners’ mental efforts and cognitive involvement when self-creating contexts rather than testing their language fluency or analyzing language forms and rules that require time to master. However, previous research suggested that appropriate contexts and accurate grammar help learners to use words well and build vocabulary knowledge since they provide understandable contextual clues for words’ semantic integration (Davidson and Ellis Weismer, 2017; Ko, 2019). It is highly conceivable that the quality of contextual clues, such as the richness of content, complexity of structure, and accuracy of grammar, would moderate EFL learners’ vocabulary acquisition when they generate contexts themselves. Further study is warranted to understand the interactive effects of internal-generated contextual clues and the quality of contexts on vocabulary learning performance.

Secondly, the present study did not investigate participants’ semantic processing on purpose when they comprehend word meanings through contextual clues due to differences in topic and focus. While EFL vocabulary acquisition relies on the understanding of contexts, enabling learners to derive word meanings through semantic relatedness (Chen et al., 2017; Joseph and Nation, 2018), it was challenging for us to ensure that every learner in the experiment could understand all example contexts due to their different prior experiences and cognitive schemas. This situation certainly occurs among EFL learners in their actual online learning processes. This leads us to a new assumption that the effects of provided contextual clues, compared to internal-generated ones, may vary based on learners’ comprehension of contexts. Previous studies have explored the correlation between specific ERPs (e.g., N400) and successful semantic processing and contextual integration when learners derive word meanings from contexts (Abel et al., 2018; Bell et al., 2019). Future work should explore how the understandability of contexts and the extent of semantic integration influence the effectiveness of contextual clues provided by materials in online vocabulary acquisition.

Finally, participants were not stratified according to their individual differences especially those relevant to language background. We did not collect their English usage background (e.g., study or travel abroad in English-speaking country) and English exposure experience (e.g., interacting with the English media and cultural products), which is positively relevant to EFL learners’ language proficiency (Lu et al., 2021; Azzolini et al., 2022). While participants all met the recruitment requirements as non-major and non-advanced learners, and the within-subjects design excluded the interference of prior knowledge, language proficiency does affect learners’ comprehension of contexts and the process of semantic integration (Yang et al., 2018). For example, learners with high proficiency tend to rely more on contextual clues to understand word meanings compared to those with lower proficiency levels (Alharbi, 2019). This kind of difference further reflects another limitation of the study regarding that various language proficiency also enables them to use different preferred strategies in language learning (Rao, 2016; Ma and Abdul Samat, 2022). The consistently employed preferred strategy facilitates vocabulary acquisition and retention for second language learners (Yang and Wu, 2015). In this case, the differences in patterns of language usage and strategies of contextual clues (e.g., preference for provided or generated contextual clues) might contribute to the observed differing vocabulary acquisition across conditions in current study. Additionally, learners who vary in language proficiency also show different attentional function when encountering target stimulus (Privitera et al., 2023). We can reasonably assume that learners with various English prior levels would perform differing attentional engagement and active attentional control when they learn lexical knowledge in videos. It is also an interesting question on the topic of online EFL vocabulary learning, which worth our exploration in further work. Further studies should be conducted to assess the feasibility of different approaches to accessing contextual clues for EFL learners with varying English proficiency levels and learning preference.

To sum up, self-generating contexts have been showed as an effective method for semantic processing and contextual integration during vocabulary comprehension, especially when compared to simply viewing contexts provided by learning materials. Future research should continue to explore the advantages and limitations of these two types of contextual clues in online EFL vocabulary learning.

## Data availability statement

The raw data supporting the conclusions of this article will be made available by the authors, without undue reservation.

## Ethics statement

The studies involving humans were approved by Ethics Committee of School of Teacher Education in Shaoxing University. The studies were conducted in accordance with the local legislation and institutional requirements. The participants provided their written informed consent to participate in this study.

## Author contributions

WZ: Writing – original draft. XW: Writing – review & editing.
